# Evaluation of Inhibitory Effect of Recreational Drugs on Dopaminergic Terminal Neuron by PET and Whole-Body Autoradiography

**DOI:** 10.1155/2014/157923

**Published:** 2014-04-29

**Authors:** Skye Hsin-Hsien Yeh, Ming-Hsien Lin, Fan-Lin Kong, Chi-Wei Chang, Li-Chung Hwang, Chien-Feng Lin, Jeng-Jong Hwang, Ren-Shyan Liu

**Affiliations:** ^1^Department of Biomedical Imaging and Radiological Sciences, National Yang Ming University, Taipei 11221, Taiwan; ^2^Department of Education and Research, Taipei City Hospital, Taipei 10341, Taiwan; ^3^Biophotonic and Molecular Imaging Research Center, National Yang Ming University, Taipei 11221, Taiwan; ^4^Division of Nuclear Medicine, Taipei City Hospital Zhongxiao Branch, Taipei 11556, Taiwan; ^5^Department of Cancer Systems Imaging, The University of Texas MD Anderson Cancer Center, TX, USA; ^6^Molecular and Genetic Imaging Core/Taiwan Mouse Clinic, National Comprehensive Mouse Phenotyping and Drug Testing Center, Taipei 11533, Taiwan; ^7^National PET/Cyclotron Center and Department of Nuclear Medicine, Taipei Veterans General Hospital, Taipei 11221, Taiwan; ^8^Nuclear Medicine Department and PET-CT Center, Shuang Ho Hospital Ministry of Health and Welfare, Taipei Medical University, New Taipei City 23561, Taiwan; ^9^Institute of Clinical Medicine, National Yang-Ming University, Taipei 11221, Taiwan

## Abstract

There is little investigation for the functional roles of peripheral dopamine. [^18^F]FDOPA has been used in cancer imaging (i.e., neuroendocrine and tumors pancreatic tumors) and neuroimaging (i.e., Parkinson's disease and Huntington's disease). Here, we accessed side effects of recreational drugs such as ketamine, cocaine, and methamphetamine on dopamine neurons in peripheral organs by using positron emission tomography (PET) imaging and quantitative whole-body autoradiography (QWBAR) with [^18^F]FDOPA. The images were applied for the measurement of specific binding ratios (SBRs) of striatum with the cerebellum as the reference region. Clear striatal [^18^F]FDOPA-derived radioactivity was observed. Moderate level of radiotracer accumulation was presented in the mucosal layers of the stomach and small intestine. The medulla layers of kidney had higher radioactivity than that of the cortex. Blocking images markedly eliminated the specific binding of [^18^F]FDOPA in the striatum and in peripheral organs such as stomachs, intestines, and kidney. Ketamine showed the highest inhibitory effect on striatal [^18^F]FDOPA-derived radioactivity followed by cocaine and methamphetamine. The current results demonstrated a useful crossing-validating tool that enhances the capability of [^18^F]FDOPA for further investigations of the alteration of dopaminergic neurons in the brain disorder or cancer diseases in peripheral tissues.

## 1. Introduction

The dopaminergic neurotransmitter system plays a crucial role in the mediation of movement cognition and emotion or neuroendocrine nature of tumor cells [1]. Small animal positron emission tomography (PET) imaging of rodent model as a key component in preclinical research and translational medicine has been used to study dopaminergic function in animal models of human diseases, including neuroendocrine tumors [2], pancreatic tumors [1], Parkinson's disease [3], Huntington's disease [4], and drug abuse [5]. These methods have been recently extended to imaging of dopaminergic function in genetically manipulated (D_2_ receptor [D_2_R] knockout) mice [6] or tumor-bearing mouse models [7].

Ketamine (2-o-chlorophenyl-2-methylamino cyclohexanone hydrochloride), as an N-methyl-D-aspartate (NMDA) noncompetitive antagonist, is a rapid-acting dissociative general anesthetic and was first used in anesthesia more than 40 years ago. Ketamine increases pain tolerance thresholds and has been shown to preserve laryngeal and pharyngeal reflexes [8]. Ironically, the very properties that restrict its clinical use have made ketamine an increasingly popular drug of abuse, to the extent that it is often erroneously sold as “Ecstasy” [9]. Indeed, ketamine has been reported to induce stereotyped behavior, a common feature of many amphetamine-type psychomotor stimulants [10].

Cocaine and methamphetamine (MA) are two of the most powerful drugs of abuse known. Up to date, there are no effective medications for these drugs abuse, dependence, or withdrawal. Despite many similar behavioral and physiological effects, MA has slower metabolism in the brain which results in it being present in the brain longer than cocaine, leading to prolonged effects [11]. Both cocaine and methamphetamine increase the levels of dopamine in the striatum; however, animal studies have shown that the levels of dopamine are higher when MA is administered [12]. Previous studies demonstrated that cocaine blocks dopamine reuptake, prolonging dopamine activity in the brain, whereas MA not only blocks dopamine reuptake, but also increases the release of dopamine. This in turn causes high dopamine concentrations in the synapse and thus longer lasting effects [12].

The role of dopamine nervous system in drug abuse and addiction has been investigated for some time [13]. The mesolimbic dopamine system, especially the nucleus accumbens (NAc), has received particular attention for its involvement in the reinforcing and addictive properties of cocaine and methamphetamine [14].

*In vivo* PET imaging using [^18^F]fluoro-3,4-dihydroxyphenyl-L-alanine ([^18^F]FDOPA), an analog of L-dihydroxyphenylalanine (L-DOPA), has been used in preclinical [15] and clinical scans of Parkinson's disease or oncology [16]. The accumulation of 6-L-[^18^F]fluorodopa in the terminals of nigrostriatal dopamine neurons reflects its transport, decarboxylation, and vesicular uptake. The uptake rate constant of 6-L-[^18^F]fluorodopa in tissue as an* in vivo* index of dopaminergic presynaptic integrity correlates well with the number surviving dopamine neurons in human subjects [17].

The effects of recreational drug such as ketamine, cocaine, and methamphetamine on dopamine neurons have been reported in the brains in rodent [18], nonhuman primate models [19], or human studies [20]; however, there is little investigation for the functional roles of peripheral dopamine or the side effects of these drugs in peripheral organs. The goal of this study was to evaluate the alteration of presynaptic dopaminergic terminal function after acute administration of ketamine by* in vivo* [^18^F]FDOPA PET imaging and quantitative whole-body autoradiography (QWBAR). In addition, we also studied groups after treatment with cocaine and methamphetamine to examine whether any observed changes were specific for ketamine. Secondly, we evaluated the roles of peripheral dopamine in metabolic homeostasis using these multi-imaging modalities.

## 2. Materials and Methods

### 2.1. ^18^F - FDOPA Synthesis

[^18^F]FDOPA synthesis was performed by use of a previously reported procedure [21]. The radiochemical purity was greater than 97% the chemical and radiochemical purities of the product isolated from the semipreparative high-pressure liquid chromatography system that were further confirmed by an analytic high-pressure liquid chromatography method (specific activity ∼18.5 ×  10^10^ Bq/mmol (5 Ci/mmol)) and were both greater than 99%. The product was made isotonic with sodium chloride and sterilized by passage through a 0.22-*μ*m Millipore filter into a sterile multidose vial.

### 2.2. Animals

Four-week-old male BALB/C mice weighing 15–20 g were used in all the studies. The animals were kept on a complete pellet diet (Lab Diet, Richmond, IN, USA) at room temperature of 25°C, with free access to tap water. The animal experiments were approved by the Laboratory Animal Care Panel of the National Yang Ming University.

### 2.3. Chemicals

Ketamine (Sin-Ton Chemicals. Taipei, Taiwan), methamphetamine (National Bureau of Controlled Drugs, Department of Health, Executive Yuan, Taipei, Taiwan), and cocaine (National Bureau of Controlled Drugs, Department of Health, Executive Yuan, Taipei, Taiwan) were used.

### 2.4. Pretreatment for [^18^F]FDOPA

Carbidopa (0.5 mg in 0.25% carbomethyl cellulose sodium salt, Sigma-Aldrich Chemicals) was given intraperitoneally 30 minutes prior to the administration of [^18^F]FDOPA.

### 2.5. Biodistribution in Mice

BALB/C mice (*n* = 3 per time point) were injected with 0.1 mL of saline solution containing 3.7 MBq of [^18^F]FDOPA through the caudal vein. Animals were sacrificed by chloroform (Nacalai Tesque Inc., Japan) at different time points from 2 minutes to 6 hours after administration of [^18^F]FDOPA. Organs of interest were removed and weighed, and the radioactivity was counted. The radioactivity concentration was determined, decay corrected, and recalculated as percentage injected dose per gram of tissue (%ID/g).

### 2.6. Quantitative Whole-Body Autoradiography (QWBAR)

The procedure has been described previously [22]. Briefly, mice were injected with 0.2 mL of a saline solution containing 3.7 MBq of [^18^F]FDOPA through caudal veins. Animals were sacrificed by chloroform at 120 minutes after injection and immediately dipped into isopentane (Nacalai Tesque Inc., Japan), which was prechilled with liquid nitrogen. The whole carcass was frozen for 1-2 minutes, depending on body size. The frozen carcass was then embedded on a cryostat holder (7 × 3 × 5 cm) with 4% carboxymethylcellulose. The embedded carcass was put on the quick freezing stage (−25°C) in the cryostat (Bright Instrument Company Ltd., UK) for about 120 minutes. Frontal sections were obtained with a Bright OTF cryomicrotome with a slice thickness of 30 *μ*m. The frozen mice were sectioned at −20°C. Pieces of prechilled adhesive tape (Deer Brand, Four Pillars Company, Taiwan) were used for lifting the frozen sections. Sections attached to tape were air dried at 22°C and awaited further processing.

For quantitative autoradiography (QAR), the tissue sections were exposed for 6–8 hours to the BAS SR-2040 phosphor imaging plate (Fujifilm) along with a set of 20-*μ*m autoradiographic standards of known ^18^F radioactivity concentration freshly prepared using pork liver homogenate. Images were acquired using a phosphorimager system FLA5100 (Fuji Photo Film Co., Tokyo, Japan). Using the known radioactivity concentration in the standards and the injected dose, the autoradiographic images were converted to color-coded parametric images of percent ID per gram tissue (%ID/g) using image analysis software MCID Elite 7.0 (InterFocus Imaging Ltd.).

### 2.7. MicroPET Imaging

Animal imaging was performed using the microPET R4 tomography (Concorde Microsystems, Knoxville, USA), which has a 12 cm animal port with an image field of view (FOV) of 11.5 cm. Animals (*n* = 3 per group or per dose of drug) were imaged at 2 hours after injection of 3.7 MBq of [^18^F]FDOPA and immediately positioned in the microPET system. Anesthesia was induced with isoflurane (4%). As soon as deep anesthesia was obtained, endotracheal intubation was performed and anesthesia was maintained by constant insufflations of 2.5% isoflurane in oxygen. Images were acquired over 30 minutes (10 min time frame × 3).

Scatter-corrected sinograms were reconstructed using the transmission scan data for ordered subset expectation maximization (OSEM). The intrinsic special resolution of the reconstructed images is 1.85 mm full width at half maximum at the center of the field of view. The image pixel size in OSEM reconstructed images was 0.4 mm transaxially with a 1.2 mm slice thickness. Radioactivity in the brains was measured in 15 brain sections.

### 2.8. Image Analysis

The striatum and cerebellum as a set of regions of interest (ROI) were drawn on the reconstructed PET images using vendor software (ASI Pro 4.1; Concorde Microsystems). Region placement was by reference to an atlas of rodent brain [23]. The cerebellum was used as the reference region assuming that no dopamine receptors are present in this region. The regional radioactivity concentrations (KBq/mL) of [^18^F]FDOPA were estimated from the maximum pixel values within each ROI expressed as percentage of injected dose/tissue g (%ID/g).

### 2.9. Blocking Experiments

For blocking studies, inhibitors were administered in various doses at 90 minutes after injection of [^18^F]FDOPA. The inhibitors and doses used for these experiments were ketamine (35, 45, or 55 mg/kg, i.p.), cocaine (5, 10, 20, or 25 mg/kg, i.v.), and methamphetamine (5, 10, 15, or 20 mg/kg, i.v.), respectively.

### 2.10. Quantification of Specific to Nonspecific Uptake Ratio of [^18^F]FDOPA in the Mice Brain

The quantification of the specific to nonspecific uptake ratio was calculated by

(1) Specific  Binding  Ratio=Radioactivity  (%ID/g)Tissue−Radioactivity  (%ID/g)ReferenceRadioactivity  (%ID/g)Reference,

where Radioactivity (%ID/g)_Tissue_ is the radioactivity concentration in the striatum and Radioactivity (%ID/g)_Reference_ is the radioactivity concentration in cerebellum.

## 3. Results

### 3.1. Biodistribution of [^18^F]FDOPA in Normal Mice

The time courses of radioactivity of [^18^F]FDOPA in brain and in various organs of the mice are shown in Table 1. The highest activity was observed in the kidney and bladder at the first 15 minutes after injection of [^18^F]FDOPA. This pattern of renal clearance was followed by fast increased radioactivity in blood and liver and subsequent clearance by kidneys. High accumulations of radioactivity were found in the liver, spleen, muscle, large intestine, and blood. Brain had the peak of radioactivity concentration in the first 30 min and then showed a gradual decrease over time; however, kidney and bladder showed a redistribution at 120 min after administration of [^18^F]FDOPA. Up to 4 hours after injection of [^18^F]FDOPA, substantial radioactivity was present in the bladder. There were less radioactivity accumulations in the heart, spleen, testis, muscle, and bone, up to 6 hours after injection.

### 3.2. *In Vivo* Imaging of [^18^F]FDOPA

**Figure 1:**
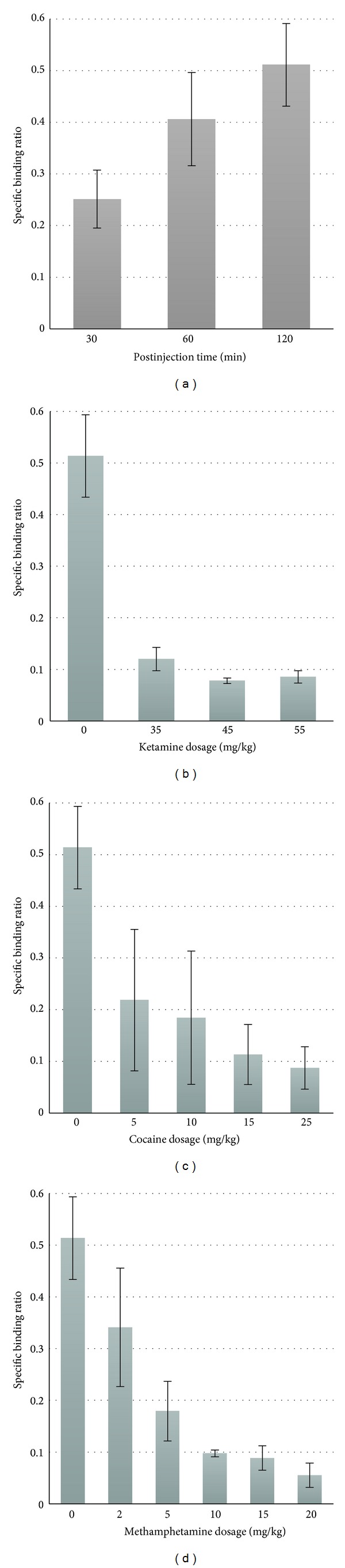
Time course of [^18^F]FDOPA radioactivity in striatum (a) and quantification of inhibitory effect of recreational drugs on the striatum radioactivity at 120 minutes after administration of [^18^F]FDOPA (b) ketamine, (c) cocaine, and (d) methamphetamine. Data shown as average ± standard error of mean (*n* = 3 per time point or per dose).

For determining pharmacokinetics of [^18^F]FDOPA in the brain of rodents, a baseline PET imaging study was performed. The time course of [^18^F]FDOPA radioactivity, normalized to the %ID/g in striatum and cerebellum of the mouse brain and then calculated for specific binding ratio (SBR), is shown in Figure 1(a). A gradually increased SBR was observed in the brain of mice with the SBR of 0.31 ± 0.026 at 30 minutes, reaching 0.48 ± 0.040 and 0.52 ± 0.008 at 60 and 120 minutes after injection, respectively. Therefore, based on the current results, we concluded that the optimal imaging time point to access SBR for [^18^F]FDOPA in mouse brain was at 120 minutes after administration of [^18^F]FDOPA. Thus, in following posttreatment studies, we acquired PET images at 120 minutes after [^18^F]FDOPA administration. In contrast, SBRs were markedly reduced by different concentrations of dopamine reuptake inhibitors, ketamine, cocaine, and methamphetamine, respectively (Figures 1(b)–1(d)). In ketamine and cocaine posttreated groups, the SBRs showed the reduction in the striatum with increasing ketamine or cocaine doses, suggesting that ketamine or cocaine inhibits the dopamine reuptake system in a dose-dependent manner resulting in a slower accumulation or inefficient utilization of [^18^F]FDOPA as an analog of L-DOPA. A similar inhibitory effect was observed in the different dosage regiment of methamphetamine (Figure 1(d)).

**Figure 2:**
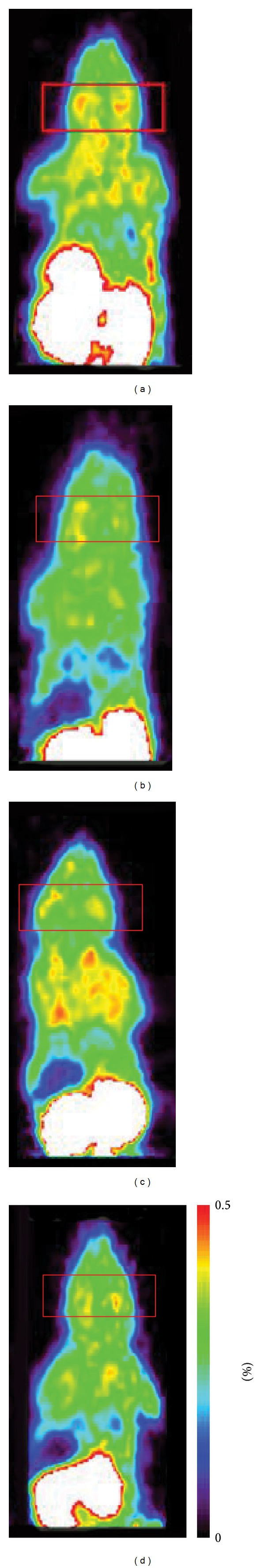
An example of axial PET images obtained 120 minutes after administration of [^18^F]FDOPA in mice showing the specific binding (a) control, following injection of a given dose of (b) ketamine (55 mg/Kg), (c) cocaine (25 mg/Kg), and (d) methamphetamine (20 mg/Kg).

PET images showed a significant accumulation of [^18^F]FDOPA in striatum at 120 min after injection (Figure 2(a)). In contrast, the level of [^18^F]FDOPA accumulation was reduced in blocking groups (Figures 2(b)–2(d)).

**Figure 3:**
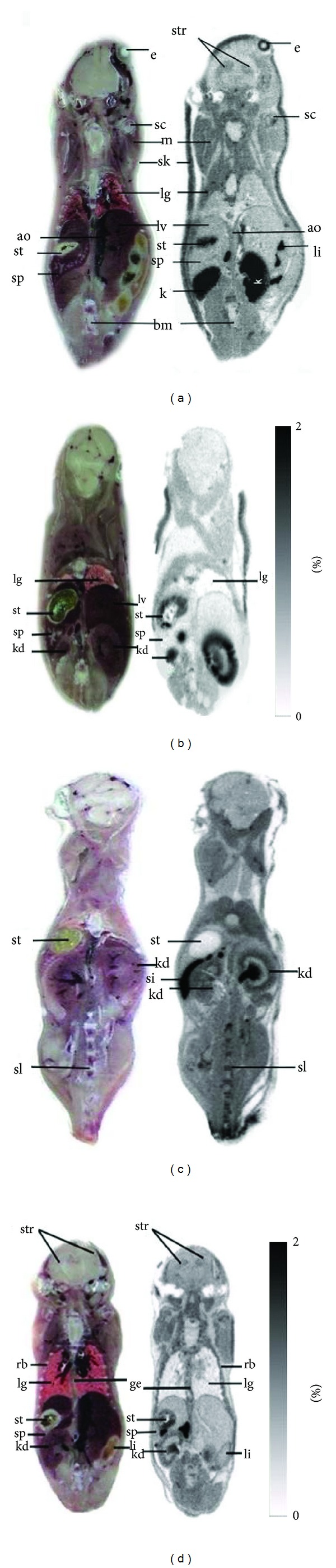
An example of axial QWBR images showing [^18^F]FDOPA distribution obtained at 120 minutes after administration of [^18^F]FDOPA (a) control, following injection of a given dose of (b) ketamine (55 mg/Kg), (c) cocaine (25 mg/Kg), and (d) methamphetamine (20 mg/Kg). ao: aorta; e: eye; str: striatum; bm: bone marrow; k: kidney; lg: lung; li: large intestine; lv: liver; m: muscle; si: small intestine; sk: skin; sp: spleen; st: stomach; rb: rib.

**Figure 4:**
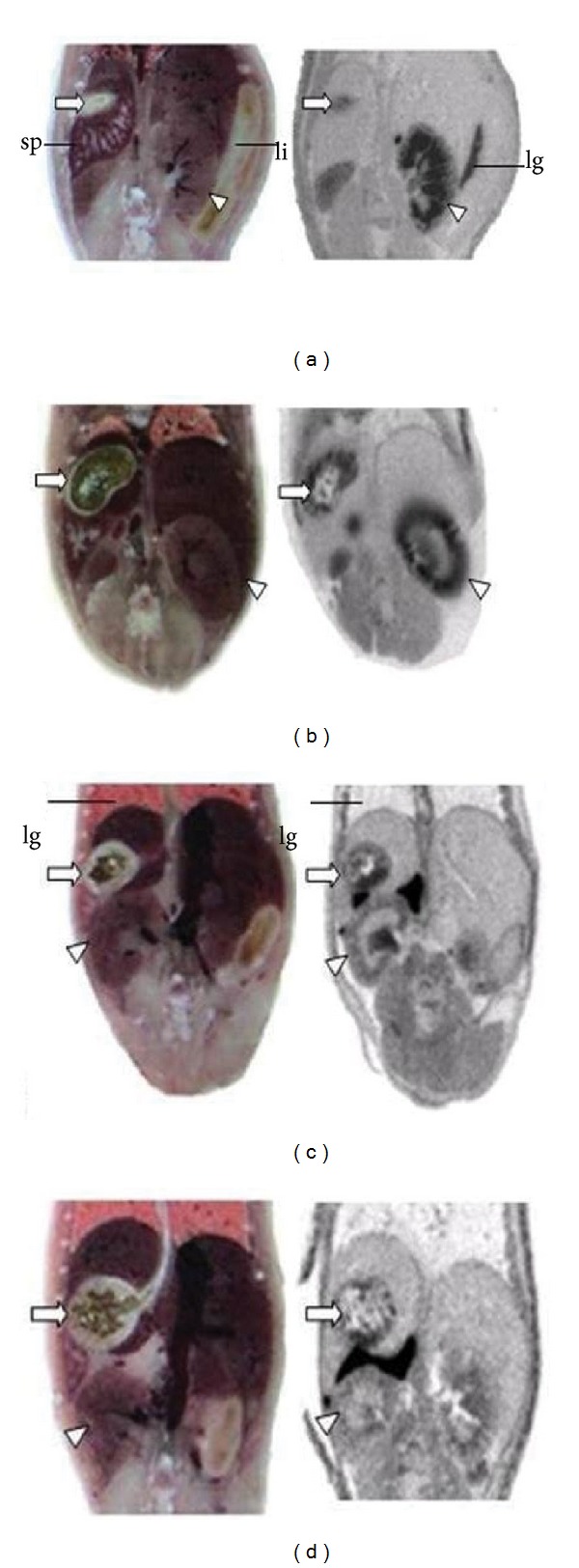
An example of magnified view of axial QWBR images of stomach and kidney in the mice obtained at 120 minutes after administration of [^18^F]FDOPA. Accumulation of the radiotracer was found mainly in the renal medulla; less or none uptake of the radiotracer can be identified in the renal cortex (triangle heads). The radiotracer was accumulated in the mucosal layer of the stomach (arrow heads) but was less accumulated in the intestine. (a) Control group, (b) ketamine (55 mg/Kg), (c) cocaine (25 mg/Kg), and (d) methamphetamine (20 mg/Kg). lg: lung; li: large intestine; k-kidney; sp: spleen; st: stomach.

In the separate group of animals (*n* = 3 per group), quantitative whole-body autoradiography (QWBAR) was performed to confirm the PET imaging results and provide anatomic details (Figure 3). At 120 min after injection of [^18^F]FDOPA, high radioactivity was observed in eye ball and aorta, which had been difficult to be distinguished by PET images (Figure 3(a)). Moderate level of radiotracer accumulation was presented in the mucosal layers of the stomach and small intestine. Little radioactivity could be identified in the brown fat, muscle, liver, and spleen. In kidneys, the medulla layers had higher radioactivity than that of the cortex. Most of the adrenal radioactivity was located in the medulla with a little found in the renal cortex region (Figure 4(a)).

Blocking images obtained from microPET and QWBAR showed marked elimination of the specific binding of [^18^F]FDOPA in the striatum. The autoradiographies demonstrated a reduction of radiotracer accumulation as compared to the control mice (Figures 3(b)–3(d)). Moreover, the significant inhibitory effect of drugs was also observed in peripheral organs such as stomachs, intestines, and kidneys (Figures 4(a)–4(d)). A summary of quantitative measures of [^18^F]FDOPA accumulation in control and blocking groups at 120 min post intravitreal injection is provided in Tables 2 and 3.

**Figure 5:**
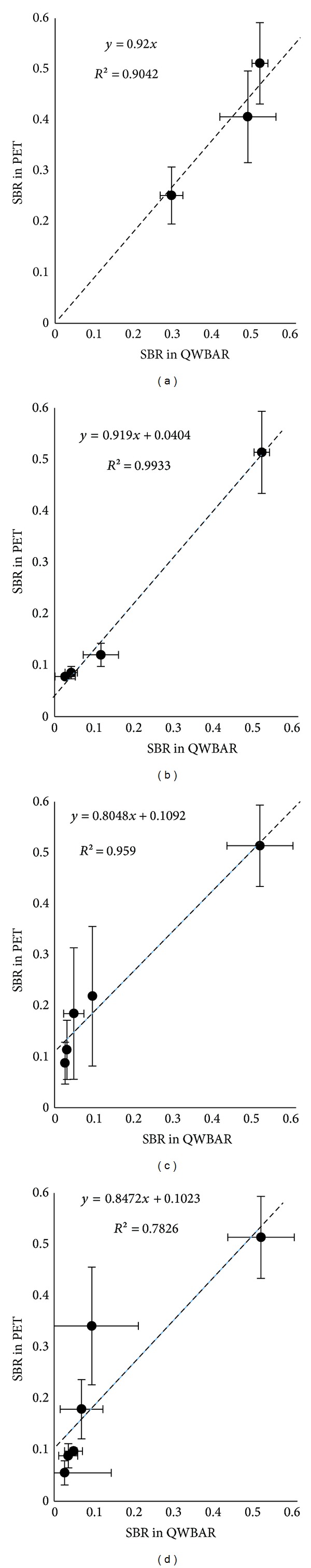
Scatter plot and linear regression analysis of relationship between the [^18^F]FDOPA PET imaging and QWBAR accumulation in striatum; an almost linear relationship is observed. (a) Control group (*r* = 0.96), (b) ketamine group (*r* = 0.99), (c) cocaine group (*r* = 0.98), and (d) methamphetamine group (*r* = 0.88).

Correlation of striatal radioactivity values obtained from both PET imaging and QWBAR yielded a Pearson correlation coefficient (*r*) = 0.9587 (Figure 5(a)). Similarly, the drugs-treated determined *r* values were 0.9061 (ketamine), 0.7864 (cocaine), and 0.9413 (methamphetamine), respectively (Figures 5(b)–5(d)). These results indicated a strong and linear relationship between PET and QWBAR images.

## 4. Discussion

This is the first study to access the* in vivo* differential alterations in peripheral dopamine by ketamine, cocaine, and methamphetamine using whole-body PET [^18^F]FDOPA and QWBAR in mice. In comparison with previous reports of [^18^F]FDOPA [15, 18], our results provided more information on tissue distribution either by tissue dissection or QWBAR and also the distribution in later time points. In the current study, despite the good SBR in striatum and cerebellum of the mice, the hepatobiliary clearance followed two-phase exponential dissociation kinetics with a fast initial phase (0–15 minutes) and a very slow subsequent phase (30–240 minutes). This biphasic pattern of liver radioactivity was reflected by a rapid increase of radioactivity concentration during the first 15 minutes in the stomach and small intestine. The renal clearance of the radiotracer was rapid and efficient and was observed by a rapid initial increase in kidney (0–15 minutes) and bladder (0–30 minutes). As the results of such pharmacokinetics of clearance, the radioactivity levels in blood rapidly dropped below 0.12% ID/g during the first 2 minutes after radiotracer injection and had exhibited characteristics of two-phase exponential clearance (Table 1). The kidney and bladder had a redistribution peak as compared with the lung and liver. The possible reason could be due to the accumulation of [^18^F]FDOPA purged by other specific target organs. Based on our results, [^18^F]FDOPA exhibited a significant hepatobiliary and renal clearances and, therefore, could reduce the radiation exposure of peripheral tissues for imaging brain at 2 hours after radiotracer administration.

We correlated the relationship among tissue-sampling biodistribution, QWBAR, and PET imaging in this study. The evidences of these positive linear relationships between variables suggested that the quantitative imaging crossing multiple-modalities could be a useful tool for preclinical research. However, in using either film or storage phosphor autoradiography as the gold standard for QWBAR, one must be aware of the fact that, because of inherent methodological problems (e.g., quenching, slight variations in slice thickness, quality of standard varying between trails with the bovine cortex, or other organs have been manually dissected and homogenized), this method is not yet supposed to yield error-free results [24, 25].

The nature of specific regional binding of [^18^F]FDOPA, predominately in the striatum region, was further validated by* in vivo* blocking studies. Virtually all of the selective binding, as indicated by the ratios of striatum/cerebellum, can be blocked by posttreatments with recreational drugs, ketamine, cocaine, and methamphetamine. We also observed the dose-dependent effect of acute administration of these drugs by using PET and QWBAR, which was in a good agreement with previous studies [18, 26, 27]. The inhibition effects of the [^18^F]FDOPA accumulation in striatum or tissues were varied. The presented data demonstrated that ketamine required higher dose than that of cocaine or methamphetamine to achieve a similar inhibition effect. This may imply that ketamine may have larger half maximal inhibitory (ID_50_) when compared to the other two; however, it needs further investigations. The explanation could be that ketamine, as an NMDA noncompetitive antagonist, can inhibit the reuptake of dopamine and increase striatal dopamine release [28, 29]. Dopaminergic response induced by ketamine results in excess dopamine into the synaptic clefts of dopaminergic neurons and then reduces the specific binding of [^18^F]FDOPA.

In present study, except for bladder, kidneys had the highest radioactivity concentration. This may raise the possibility of extra radiation burden in the kidney tissues contributed by the radio-metabolites. The potential damage to the organ caused by the extra radiation should be avoided as possible unless only small amount of radiotracer is applied.

On the other hand, it was noted that relatively higher inhibitory effect of recreational drugs was found in renal medulla when compared to other peripheral tissues, suggesting a mechanism by which recreational drugs abuse may cause urinary tract abnormalities. Despite valuable properties as an anesthetic agent, the use of ketamine has been limited by extent and variety of side effect. There is growing evidence that chronic use or abuse of ketamine is linked with major urological syndrome such as inflammatory cystitis with low volume bladders. Selby et al. reported a clinical case that the inflammatory cystitis and reversible hydronephrosis of ketamine abuse were associated with the precipitation of ketamine metabolites in the ureters and acute renal failure [30]. By using a mouse model, Yeung et al. strengthened the linkage between ketamine addition and urinary tract damage [31]. These findings may also have implications for the drug's use as an antidepressant [32]. Autry et al. showed that in mouse models of depression, ketamine promotes the rapid synthesis of brain-derived neurotrophic factor (BDNF) and p-mTOR that is known to have antidepressant effects, suggesting that this may provide a therapeutic target for developing fast-acting antidepressants [32]. Moreover, Walker et al. developed the means to assess the uptake and trapping rate of dopamine by [^18^F]FDOPA and the rate constant for the loss of [^18^F]FDOPA metabolites which enhances the capability of [^18^F]FDOPA imaging to assist in the development of novel therapies in brain disorder associated with dopaminergic function [33].

Carbidopa, a competitive inhibitor of aromatic l-amino acid decarboxylase (AADC), is routinely used prior to [^18^F]FDOPA administration in clinical or preclinical study of neuroimaging in order to minimize the peripheral metabolism and to increase radio concentration of in the brain. Carbidopa increased the accumulation of [^18^F]FDOPA in sham and tumor-bearing mice three- or fourfold, respectively, when compared to vehicle pretreatment. However, there was no significant difference between Carbidopa pretreated sham and tumor-bearing mice [1]. Therefore, we concluded that the effect of Carbidopa in brain and peripheral organs could be neglected since we pretreated all animal with Carbidopa in the same condition and compared the accumulation of [^18^F]FDOPA as % inject dose per tissue gram before and after dosing with recreational drugs.

Recently, increasing evidence has implicated ketamine that induces depression of excitatory synaptic transmission in the nucleus accumbens [34]. Also, ketamine causes the alterations of histone deacetylase (HDAC) activity and (BDNF) brain-derived neurotrophic factor in the prefrontal cortex, hippocampus, amygdala, and nucleus accumbens [35]. The current findings support the role of epigenetic mechanisms in nucleus accumbens that contributes to transcriptional and behavioral changes induced by recreational drugs. It motivates that coupled-imaging through PET [^18^F]FDOPA and [^18^F]FAHA, a radiotracer for HDAC activity, could be used to evaluate the withdrawal phase which is associated with gene-specific changes in acetylation of histone protein as well as development of antidepressants without these negative effects.

## 5. Conclusion

The current results provided a useful crossing-validation tool for the further understanding of the alteration of dopaminergic neurons in the brain or peripheral organs. These findings indicated that the [^18^F]FDOPA imaging along or coupled with [^18^F]FAHA could be a promising means for assessment the malfunction of peripheral dopamine in tissues including the brain caused by recreational drugs or evaluation of therapy efficiency of antidepressants.

## Figures and Tables

**Table 1:** Biodistribution of accumulated radioactivity (%ID/g) in different tissues at the designed time points after administration of [^18^F]FDOPA in normal mice.

Radioactivity (% injection dose/tissue g)						
Organs	2 min	15 min	30 min	60 min	120 min	240 min
Skin	0.06 ± 0.030	0.44 ± 0.140	0.47 ± 0.220	0.36 ± 0.510	0.33 ± 0.070	0.31 ± 0.060
Blood	0.12 ± 0.000	0.62 ± 0.170	0.58 ± 0.110	0.45 ± 0.080	0.31 ± 0.060	0.13 ± 0.040
Heart	0.07 ± 0.020	0.63 ± 0.270	0.51 ± 0.020	0.43 ± 0.060	0.28 ± 0.040	0.16 ± 0.050
Spleen	0.04 ± 0.010	0.73 ± 0.130	0.65 ± 0.160	0.44 ± 0.050	0.40 ± 0.110	0.19 ± 0.070
Stomach	0.28 ± 0.090	1.07 ± 0.300	1.15 ± 0.080	0.91 ± 0.150	0.78 ± 0.170	0.47 ± 0.060
Liver	0.06 ± 0.040	0.87 ± 0.210	0.87 ± 0.160	058 ± 0.180	0.34 ± 0.090	0.16 ± 0.060
Small intestine	0.09 ± 0.030	0.87 ± 0.200	1.01 ± 0.210	1.13 ± 0.190	0.56 ± 0.220	0.20 ± 0.040
Large intestine	0.05 ± 0.020	0.57 ± 0.170	0.60 ± 0.320	0.49 ± 0.110	0.39 ± 0.040	0.21 ± 0.050
Kidney	0.18 ± 0.060	4.07 ± 1.880	2.84 ± 0.230	2.84 ± 0.560	4.14 ± 0.100	3.25 ± 0.700
Bladder	0.04 ± 0.020	3.75 ± 2.240	7.88 ± 5.220	4.51 ± 1.850	11.25 ± 3.960	2.30 ± 0.850
Testis	0.02 ± 0.010	0.37 ± 0.180	0.40 ± 0.080	0.28 ± 0.020	0.26 ± 0.260	0.16 ± 0.100
Muscle	0.03 ± 0.020	0.50 ± 0.310	0.60 ± 0.370	0.45 ± 0.050	0.45 ± 0.60	0.19 ± 0.080
Bone	0.04 ± 0.020	0.46 ± 0.250	0.43 ± 0.140	0.33 ± 0.040	0.40 ± 0.030	0.19 ± 0.060
Brain	0.02 ± 0.000	0.36 ± 0.180	0.38 ± 0.040	0.31 ± 0.040	0.23 ± 0.040	0.09 ± 0.030

Data shown as average ± standard error of mean (*n* = 3 per time point).

**Table 2:** Accumulation of [^18^F]FDOPA in different imaging modalities in mice.

Control	PET	QWBAR
30 min	0.251 ± 0.056	0.290 ± 0.028
60 min	0.406 ± 0.090	0.480 ± 0.070
120 min	0.511 ± 0.080	0.523 ± 0.020

Data shown as average ± standard error of mean (*n* = 3 per dose).

**Table 3:** Accumulation of [^18^F]FDOPA radioactivity in different imaging modalities in mice after treatment with recreational drugs.

Ketamine	PET	QWBAR
0 mg/kg	0.511 ± 03080	0.523 ± 0.082
35 mg/kg	0.120 ± 0.022	0.114 ± 0.044
45 mg/kg	0.078 ± 0.005	0.026 ± 0.025
55 mg/kg	0.085 ± 0.012	0.041 ± 0.015

Cocaine	PET	QWBAR

0 mg/kg	0.511 ± 0.080	0.523 ± 0.085
5 mg/kg	0.218 ± 0.137	0.094 ± 0.003
10 mg/kg	0.184 ± 0.129	0.048 ± 0.025
15 mg/kg	0.113 ± 0.058	0.031 ± 0.004
25 mg/kg	0.087 ± 0.041	0.026 ± 0.001

Methamphetamine	PET	QWBAR

0 mg/kg	0.511 ± 0.080	0.523 ± 0.082
2 mg/kg	0.341 ± 0.114	0.093 ± 0.115
5 mg/kg	0.179 ± 0.058	0.068 ± 0.053
10 mg/kg	0.097 ± 0.006	0.048 ± 0.022
15 mg/kg	0.088 ± 0.023	0.035 ± 0.023
20 mg/kg	0.055 ± 0.023	0.026 ± 0.115

Data shown as average ± standard error of mean (*n* = 3 per dose).
